# Benign and Malignant Nodular Thyroid Disease in Acromegaly. Is a Routine Thyroid Ultrasound Evaluation Advisable?

**DOI:** 10.1371/journal.pone.0104174

**Published:** 2014-08-15

**Authors:** Jordi L. Reverter, Carmen Fajardo, Eugenia Resmini, Isabel Salinas, Mireia Mora, Mariona Llatjós, Gemma Sesmilo, Ferran Rius, Irene Halperin, Susan M. Webb, Veronica Ricart, Pedro Riesgo, Dídac Mauricio, Manel Puig-Domingo

**Affiliations:** 1 Service of Endocrinology and Nutrition, Department of Medicine, Germans Trias i Pujol Health Science Research Institute and Hospital, Universitat Autònoma de Barcelona, Badalona, Spain; 2 Service of Endocrinology, Hospital Universitario de la Ribera, Alzira, Spain; 3 IIB- Sant Pau and Service of Endocrinology, Department of Medicine, Centro de Investigación Biomédica en Enfermedades Raras (CIBER-ER Unidad 747), Hospital Sant Pau, Universitat Autònoma de Barcelona, Barcelona, Spain; 4 Service of Endocrinology, Hospital Clínic, Barcelona, Spain; 5 Service of Pathology, Department of Medicine, Germans Trias i Pujol Health Science Research Institute and Hospital, Universitat Autònoma de Barcelona, Badalona, Spain; 6 Service of Endocrinology, Hospital Universitari Quiron-Dexeus, Barcelona, Spain; 7 Service of Endocrinology, Hospital Universitari Arnau de Vilanova, Lleida, Spain; 8 Service of Radiology, Hospital Universitario de la Ribera, Alzira, Spain; 9 Service of Neurosurgery, Hospital Universitario de la Ribera, Alzira, Spain; University of Nebraska Medical Center, United States of America

## Abstract

Data on the prevalence of benign and malignant nodular thyroid disease in patients with acromegaly is a matter of debate. In the last decade an increasing incidence of thyroid cancer has been reported. The aim of this study was to evaluate the prevalence of goiter, thyroid nodules and thyroid cancer in a large series of patients with acromegaly with a cross-sectional study with a control group. Six Spanish university hospitals participated. One hundred and twenty three patients (50% men; mean age 59±13 years; disease duration 6.7±7.2 years) and 50 controls (51% males, mean age 58±15 years) were studied. All participants underwent thyroid ultrasound and fine needle aspiration. Cytological analysis was performed in suspicious nodules between 0.5 and 1.0 cm and in all nodules greater than 1.0 cm. Goiter was more frequently found in patients than in controls (24.9 *vs.* 8.3%, respectively; p<0.001). Nodular thyroid disease as well as nodules greater than 1 cm were also more prevalent in acromegalic patients (64.6%, *vs.* 28.6%, p<0.05 and 53.3 *vs.* 28.6%, respectively; p<0.05), and all underwent fine needle aspiration. Suspicious cytology was detected in 4 patients and in none of the controls. After thyroidectomy, papillary thyroid carcinoma was confirmed in two cases (3.3% of patients with thyroid nodules), representing 1.6% of the entire group of patients with acromegaly (2.4% including a case with previously diagnosed papillary thyroid carcinoma). These data indicated that thyroid nodular disease and cancer are increased in acromegaly, thus justifying its routine ultrasound screening.

## Introduction

Acromegaly is an endocrine disorder characterized by growth hormone (GH) hypersecretion mainly due to a pituitary micro or macroadenoma [1]. In the majority of patients GH excess produces an overgrowth of acral parts of the body. Organs such as the liver or the heart may also increase in size and joint articular cartilage thickness, vertebral fractures, left ventricular dysfunction, abnormal lipid profile, and obstructive apnea events may appear with different reported prevalence [2]. Moreover, long term exposure to GH and insulin-like growth factor (IGF)-1 could induce proliferative capacity and increases the likelihood of developing malignancies [3], colorectal cancer being the most frequently observed neoplasm in acromegalic patients. Therefore, colonoscopy is recommended for early detection and removal of pre-malignant intestinal polyps and colorectal cancer [4].

Nodular thyroid disease and goiter are frequent conditions in the general population, with an age-related increasing incidence reaching 30–50% in people over 50 years in ultrasound studies [5]. Most of these nodules are benign, but the overall reported malignancies rate is about 5–10% [6]. The age-standardized incidence of thyroid cancer is estimated to be 0.9% (females) and 0.2% (males) in developed countries and related to iodine deficiency status [7].

The prevalence of benign nodular thyroid disease and malignancy in patients with acromegaly has been a matter of debate. In previous studies performed many years ago [8]–[11], goiter and thyroid nodules appeared to be more frequent among patients with acromegaly, but the prevalence of thyroid carcinoma was thought to be low, and its true incidence was unknown. More recent studies [12]–[15] have reported an overwhelming increase in thyroid cancer prevalence (11.0, 7.2, 7.8 and 10.6%, respectively). However, only the one by dos Santos *et al.* [13] evaluated a significantly large number of patients in a cross-sectional protocol, to be considered consistent enough. The other studies from the same institution [14], [15], are retrospective [14], [15] and included a limited number of patients [12], [14], [15].

Thus, the true prevalence of thyroid nodules and thyroid cancer in acromegaly warrants further assessment, with more studies in different geographical areas and including a contemporary control group.

Therefore, our objective was to evaluate the prevalence of goiter, thyroid nodules and thyroid cancer in a large series of acromegaly patients in Spain, compared to an age- and sex-matched control group.

## Materials and Methods

### Patients

We conducted a multicenter cross-sectional study including a control group. Patients followed for acromegaly were recruited from the outpatient clinics of six university hospitals in Spain: Santa Creu i Sant Pau University Hospital, Clinic University Hospital and Hospital Universitari Quiron-Dexeus, all in Barcelona, Germans Trias i Pujol University Hospital in Badalona, Arnau de Vilanova University Hospital in Lleida, and Hospital de la Ribera in Alzira, Valencia. All these hospitals are located in non-iodine deficient areas [16]. The control group was composed by otherwise healthy subjects over 18 years-old. The study was conducted in accordance with the Declaration of Helsinki and was approved by the relevant local Human Research Ethics Committee (Comité d'Ètica de la Investigació Clínica, CEIC, Hospital Germans Trias i Pujol de Badalona). All participants gave written informed consent.

### Methods

All patients actively followed-up in the different institutions were invited to participate. From an original cohort of 140 patients, 123 accepted the invitation. Seventeen were not included due to non-medical reasons. Diagnosis of acromegaly was confirmed when elevated IGF-1 levels (adjusted for sex and age) and GH levels greater than 1 ng/mL after an oral glucose tolerance test with 75 g of dextrose and a pituitary adenoma identified by conventional imaging techniques. All patients were treated following institutional protocols and international guidelines for acromegaly management, with surgery, medical therapy (somatostatin analogues, cabergoline or pegvisomant) and external radiation when needed [4], [17], [18]. Disease duration, time from first symptoms, age at diagnosis, treatment modalities and history of thyroid disorders were recorded for each patient. Duration of disease was considered from the date of diagnosis to evaluation.

Serum GH levels were locally measured in each participating center by chemiluminescent assay and serum IGF-1 levels were measured at each site by immunometric chemiluminescent immunoassay; serum free thyroxine (fT4) and TSH were measured by electrochemiluminescence immunoassay (Siemens, Los Angeles, CA, USA).

In each patient an ultrasound examination was performed using a 7.5–12 MHz linear transducer device by experienced operators (JLR, VR) in two centres using a common protocol. Morphological evaluation included the description of thyroid echostructure and the measurement of the diameters of each thyroid lobe and isthmus. Volumetric assessment of the thyroid gland was based on the use of an ellipsoid model [19]. With this rotating ellipsoid model, the height, width, and depth of each lobe were measured and multiplied. The obtained result was then multiplied by the mathematical constant or correction factor 0.524 [20]. The result is the estimated value of the volume of each lobe and the isthmus; this procedure is also performed for determining the volume of the isthmus. Thus, calculation of the total volume of the gland results from the sum of the volume of each lobe and the volume of the isthmus. According to the reference values obtained in the population in our area, the mean normal gland volume of an adult individual has been estimated to be 9.19 mL (CI: 9.87–10.65 mL) in men and 6.19 mL (CI: 6.22–6.92 mL) in women [21].

All the nodules detected were measured and their ultrasound characteristics were registered; cervical lymph nodes were also evaluated. Fine-needle aspiration (FNA) cytology was performed in all nodules larger than 1.0 cm of diameter and in those between 0.5 and 1.0 cm if they presented suspicious ultrasound characteristics according to the American Society of Ultrasonography [22], such as the presence of microcalcifications, irregular borders, increased central flow on Doppler examination, taller than wide diameter, hypoechogenicity and absence of halo. FNA was performed using a 25 GA needle (Becton, Dickinson and Company, Sparks, MD, USA), and guided by ultrasound in two centres (JLR,VR) using the same protocol. The slides were air-dried, and then stained with Giemsa. Two pathologists evaluated the samples. The nodules with a suspicious or firm diagnosis of thyroid carcinoma in FNA cytology were referred for thyroidectomy.

### Statistical analysis

Continuous variables were expressed as mean ± SD and categorical variables as percentages. Student's t test was used for comparisons between continuous variables and Pearson's correlation test for correlation analyses. A p value <0.05 was considered statistically significant and correlations were considered significant for Pearson's correlation coefficient if ≥0.25. For comparison of categorical variables, the Chi-squared test or the Fisher exact test were used where appropriate. Data analyses were performed using Statistical Package for Social Sciences (SPSS^©^, Chicago, IL, USA) for Windows, Version 15.0.

## Results

### Subjects characteristics

The subjects of this study were 123 acromegalic patients (50% males), with a mean age of 59±13 years. Time between initial symptoms and diagnosis was 5.4±5.2 years. Disease duration from diagnosis was 6.7±7.2 years. Family history revealed goiter or thyroid disease in 2.9% of the patients. Nine cases had undergone previous thyroid surgery for well documented benign nodular goiter and one case for papillary thyroid carcinoma. A GH producing macroadenoma was the cause of acromegaly in 73.4% of the cases and in the remaining 16.6% a microadenoma. Ninety seven percent underwent neurosurgical treatment, and external radiotherapy was performed in 36.6%.

With regards to disease status at the time of the present study, 51.4% of patients still had active disease and were under medical therapy. Of those: 63.4% were treated with long-acting somatostatin analogues, 29.6% with cabergoline and 27.8% with pegvisomant. Table 1 shows clinical and analytical characteristics of patients with active and cured disease. Last serum IGF-1 was significantly lower in cured patients compared to non-cured (197.2±72.0 ng/mL *vs.* 258.1±147.5 ng/mL, respectively, p = 0.02). The 52.5% of patients had no associated pituitary hormone deficiencies, whereas the rest required one (37.5%) or multiple (10%) substitution therapies.

**Table 1:** Characteristics of patients with cured acromegaly and active disease.

	Cured (n = 63)	Active (n = 60)	p value
**Age (years)**	60±11	58±14	0.4
**Initial GH (µg/L)**	22.4±44	20.7±21.6	0.8
**Initial IGF-1 (ng/mL)**	769.1±413.5	939.0±460.6	0.1
**Last IGF-1 (ng/mL)**	197.2±72.0	258.1±147.5	0.02
**TSH** * **(mU/L)**	2.0±1.7	1.4±1.2	0.1
**Free T4 (ng/dL)**	1.1±0.3	1.2±0.2	0.6

* Patients without TSH deficiency.

GH: Growth hormone, IGF: Insulin-like growth factor. TSH: Thyrotropin.

The control group was composed of 50 volunteers (51% males) with a mean age of 58±15 years.

### Ultrasound characteristics

**Figure 1:**
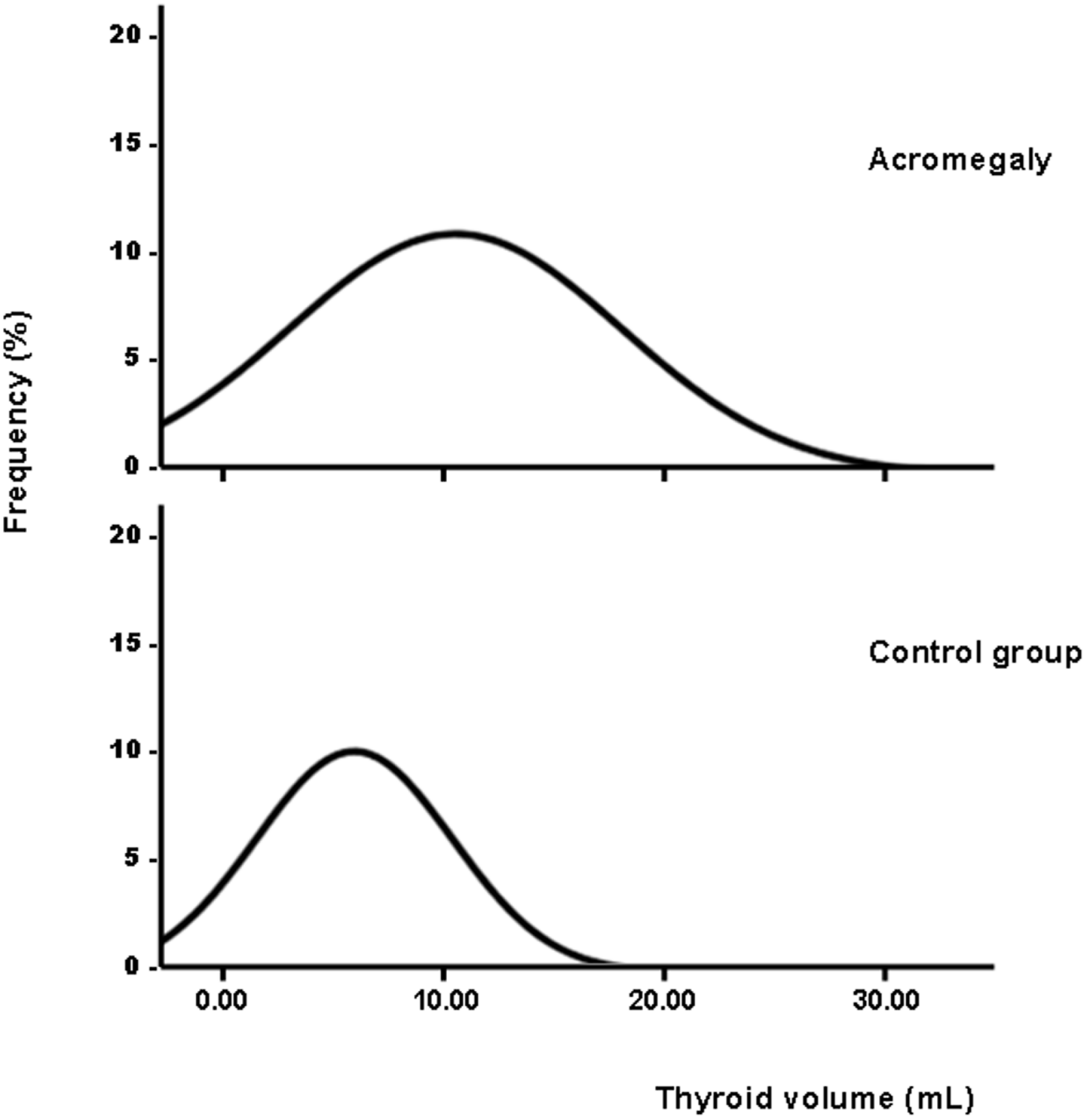
Thyroid volume (mL) in acromegalic patients (upper panel) and controls (lower panel) following a normal distribution.

The results of ultrasound examination and FNA are depicted in Table 2. Thyroid volumes obtained in the control group were similar to those previously observed in a population of the same geographical area with a similar iodine intake [21]. The overall group of acromegalic patients showed an increased thyroid volume compared to the control group (12.6±7.5 *vs.* 8.0±4.5 mL, p<0.001). This difference was also observed when comparing male and female patients with their respective controls. Acromegalic male patients presented non-significant greater thyroid volume compared with females (13.0±6.3 ml *vs.* 12.2±8.9 ml, respectively, p = 0.6). Goiter was more frequently observed in patients compared to controls (24.9 *vs.* 8.3%, respectively; p<0.001). As shown in Figure 1, thyroid volume values presented a normal distribution in both groups. On the other hand, patients with active acromegaly showed larger thyroid volumes than those with cured disease (14.6±8.7 *vs.* 10.9±5.3 mL, respectively; p = 0.03).

**Table 2:** Sonographic features and cytological diagnosis of patients with acromegaly and controls group.

	Acromegaly (n = 123)	Controls (n = 50)	p value
**Thyroid volume (mL) (mean±SD)**			
**- Overall group**	12.6±7.5	8.0±4.5	<0.001
**- Male**	13.0±6.3	8.8±5.7	<0.05
**- Female**	12.2±8.9	7.0±2.5	<0.05
**Thyroid nodules (%)**	55.7	30.0	<0.05
**- Solitary**	25.5	16.7	<0.05
**- Multiple**	30.2	12.5	<0.05
**Nodule diameter <1 cm (%)**	44.7	71.4	<0.05
**FNA** * **cytology (% of patients with nodules)**	55.2	28.6	<0.001
**Diagnosis (% of smears**			
**- Benign**	87.0	100.0	<0.05
**- Suspicious for a follicular neoplasm**	6.5	0.0	<0.05
**- Malignant (papillary carcinoma**	6.5	0.0	<0.05

* FNA: Fine needle aspiration.

Newly discovered nodular thyroid disease was more prevalent in acromegaly (55.7%), while nodules were detected in one third of the controls (30.0%, p<0.05). The relative distribution of single and multiple nodular thyroid disease was different as well, with more multinodular goiter in acromegalic patients. The overall prevalence of thyroid nodules in our acromegalic patients reached 64.6% including patients with previously known nodular thyroid disease.

The size of the nodules was significantly different between patients and controls. Micronodules <1 cm were detected more frequently in controls (71.4%) than in acromegalic patients (44.7%, p<0.05) who therefore presented a higher prevalence of clinically significant thyroid nodules. In acromegalic patients, 5% of the micronodules smaller than 1 cm diameter had suspicious sonographic features that led to FNA and in all of them the cytological exam was benign (colloid goiter and thyroid cyst). On the other hand, none of the micronodules in controls required cytological evaluation.

### Cytological and histopathological diagnosis

FNA was performed in 55.2% of patients with thyroid nodules and in 28.6% of the controls (p<0.001). As shown in Table 2, the majority of smears of acromegalic patients were diagnosed as benign lesions, especially colloid goiter, and to lesser extent thyroid cysts. Suspicious or malignant cytology was detected in 13.0% of the FNAs specimens from acromegalic patients. All these patients with suspicious or positive malignant cytology underwent thyroidectomy. Papillary thyroid carcinoma was confirmed in two cases, representing 3.2% of patients harbouring thyroid nodules (1.6% of the overall acromegaly group). The overall prevalence of thyroid carcinoma, including the patient with previously diagnosis of papillary thyroid cancer was 2.4%.

None of the nodules cytologically analyzed in the control group was suspicious or malignant.

### Correlation studies

In acromegaly, no correlation was found between thyroid volume and the duration of the disease, time between the first symptoms to diagnosis, GH and IGF-1 at diagnosis, and IGF-1 at last follow-up. Patients with thyroid nodular disease and those with normal ultrasound presented similar concentrations of GH (24.±43.0 *vs.* 27.1±29.4 µg/L, respectively; p = 0.4) and IGF-1 (867.5±438 *vs.* 825.8±525.8 ng/mL, respectively; p = 0.7) at diagnosis. IGF-1 concentrations at last follow-up were not different in patients with thyroid nodules (226.3±144.8 ng/mL) compared to patients without thyroid abnormalities (239.7±139.6 ng/mL) (p = 0.7).

Bivariate correlation studies showed that age was positively correlated with TSH (Rs = 0.2; p = 0.02) whereas thyroid volume and nodule size were not correlated with TSH, GH or IGF-1 concentrations.

The two cases of newly diagnosed papillary thyroid carcinoma were observed in active acromegalic patients (one male, one female), one with a macroadenoma and the other with a microadenoma diagnosed in 2011 and 2012, respectively. In these two patients, initial IGF-1 levels were 1453.1 and 533.3 ng/mL, respectively and the size of their nodules was greater than 2 cm. The case with a previous diagnosis of papillary thyroid cancer (2 cm diameter) was a male with a pituitary macroadenoma, diagnosed in 2013 with initial levels of IGF-1 of 894.2 ng/mL.

## Discussion

In our series of 123 acromegalic patients accurately explored for thyroid abnormalities, we found an increased prevalence of goiter and thyroid nodules and three cases of thyroid cancer. This rate of thyroid malignancy (2.4%) is lower than previously reported [12]–[15], but represents a significant increase with respect to the general population [23], [24]. However, these figures should be interpreted in parallel to epidemiological data from specific geographical regions. In this sense, reported thyroid cancer incidence and prevalence varies considerably in different registries [25]–[27].

Earlier studies with smaller number of cases and recent data from a large series of patients reported an overall detection of goiter of about 20% in patients with acromegaly [13], [28]. The present study, confirms a rate of goiter in acromegalic patients of 24.9%, higher than in the control group (8.3%). Interestingly, as a group, thyroid volume of the patients followed a normal distribution, suggesting a generalized effect. Thyroid size was not related to age or to other clinical and laboratory parameters. However, as expected, TSH levels in patients not treated with thyroxine correlated positively with age, but were within the normal range (data not shown) [29]. The increased thyroid volume in acromegaly is likely to be a consequence of continuous stimulation by excessive secretion of GH from the pituitary adenoma. However, unlike previous studies [14], no correlation was found between GH or IGF-1 levels at baseline or last follow-up and thyroid volume. We have not a clear explanation for this negative finding, but probably individual factors play a role in the response to GH stimuli. As normal thyroid volume varies depending on the presence of iodine deficiency, differences in iodine supplies could also have influenced thyroid growth [30].

The prevalence of thyroid nodules of 55.7% in our study is similar to that reported by others with a similar number of studied cases (54%) [13] and is even more pronounced (64.6%) when considering patients with a previous diagnosis of nodular thyroid disease. However, in comparison to previous reports [13], [14], we found more nodules of a diameter greater than 1 cm and for this reason more FNA cytological evaluations have been performed, the majority being benign. As expected in the general population, nodular thyroid disease was detected in about one third of controls, similar to data from other European epidemiological studies [31], with no thyroid cancer found. Iodine intake in our population [30] could influence this proportion of thyroid nodules. On the other hand, a higher number of micronodules (<1 cm) were observed in controls than in patients with acromegaly. Prolonged exposure to high levels of GH and/or IGF-1 may be the basis of this increase in the frequency and size of the nodules.

Growth hormone and IGF-1 have both well known proliferative and anti-apoptotic effects and their hypersecretion may theoretically induce tumor development and stimulate its growth. Although the influence of acromegaly on carcinogenesis remains controversial, several studies indicate that the rate of tumors in these patients is higher. In this regard, acromegaly has been associated with both benign and malignant tumors, and carcinoma is the third cause of death in acromegalic patients [18]. Therefore, active detection of colon cancer is recommended in international guidelines [4]. Regarding thyroid cancer, this issue continues to be controversial so far [32] and, specific recommendations for ultrasound screening have not been included in recent guidelines [4]. As thyroid follicular cells express IGF-1 receptors and IGF-1 is a well recognized growth factor for thyrocites, it may be speculated that IGF-1 has a potential role in the development of thyroid cancer in acromegalic patients. However, no difference was found in GH/IGF-1 concentrations in patients with and without thyroid cancer. On the other hand, IGF-1 receptor expression in thyroid cancer samples was not evaluated. Individual sensitivity, epigenetic modifications, different patterns of GH/IGF-1 secretion over time and other factors yet unknown could explain this lack of differences.

At present, due to the improvement in surgical and radiotherapeutic procedures [33], [34] as well as the advances in medical treatment [35], survival of patients with acromegaly has substantially improved. Therefore, the appearance of slow-growing tumors that could compromise quality of life or survival is a reasonable possibility dueto the frequently long delay in the diagnosis of acromegaly, causing long exposure to high GH levels.

Thyroid carcinoma is generally characterized by its low aggressiveness [23], [24]. However, between 15 and 25% of cases may undergo a dedifferentiation process with loss of the ability to trap radioiodine, making this treatment ineffective, and worsening the prognosis [36], [37].

Few studies have focused on the incidence of thyroid cancer in acromegaly using ultrasound examination and FNA biopsy [13]–[15]. Among these, only one has been able to include more than one hundred patients with a multicenter design [13]. A feature of more recent reports, two retrospective studies from the same Turkish group [14], [15] and one cross-sectional from Brazil, is the remarkable high prevalence of thyroid cancer found, which reached 7.8% [14], 10.6% [15] and 7.2% [13]. In our cases we would have expected at least 7 to 12 thyroid carcinomas. However, the diagnosis of two new cases of papillary thyroid cancer in our series is far from these previous observations. This discrepancy may be due to geographical, ethnic or environmental reasons such as iodine intake or the prevalence of thyroid autoimmunity. Even so, the prevalence found in the present study represents a significant increase with respect to the general population. Moreover newly discovered nodular thyroid disease was more prevalent in acromegalic patients, with a higher prevalence of goiter. These data support, in our opinion, the recommendation for routine thyroid ultrasound for systematic detection of thyroid cancer in acromegaly and FNA cytology when indicated.

In a recent meta-analysis and systematic review in this topic [38], the authors state that the amount of reliable papers including controls groups and data of both on the prevalence of thyroid nodular disease and thyroid cancer is unsatisfactory. The main strengths of our study are the non-retrospective design, the sample size achieved by the multicenter approach, the systematic exploration of thyroid gland by using high sensitive ultrasound devices and the standardized defined nodule characteristics for FNA cytology. Furthermore, to our knowledge, this is the largest study in this issue performed in Western European countries in the last decade after the Italian multicenter study [8]. Locally determined GH and IGF-1 in each participating center may influence the correlation studies, but not the results referring to thyroid nodules and cancer prevalence. The number of the control subjects is adequate to make a conclusion about thyroid volume and goiter prevalence but could be insufficient for detection of thyroid cancer. However, this fact does not influence the objective assessment of the number of malignant tumors found in the patients.

In conclusion, thyroid nodular disease and thyroid malignancy are increased in acromegaly compared to the general population, thus justifying its systematic ultrasound screening.
